# Replication of an empirical approach to delineate the heterogeneity of chronic unexplained fatigue

**DOI:** 10.1186/1478-7954-7-17

**Published:** 2009-10-05

**Authors:** Eric Aslakson, Uté Vollmer-Conna, William C Reeves, Peter D White

**Affiliations:** 1 Centers for Disease Control and Prevention, Atlanta, Georgia, USA; 2 School of Psychiatry, University of NSW, Sydney, Australia; 3 Center for Psychiatry, Wolfson Institute of Preventive Medicine, Barts and the London School of Medicine, Queen Mary University of London, UK

## Abstract

**Background:**

Chronic fatigue syndrome (CFS) is defined by self-reported symptoms. There are no diagnostic signs or laboratory markers, and the pathophysiology remains inchoate. In part, difficulties identifying and replicating biomarkers and elucidating the pathophysiology reflect the heterogeneous nature of the syndromic illness CFS. We conducted this analysis of people from defined metropolitan, urban, and rural populations to replicate our earlier empirical delineation of medically unexplained chronic fatigue and CFS into discrete endophenotypes. Both the earlier and current analyses utilized quantitative measures of functional impairment and symptoms as well as laboratory data. This study and the earlier one enrolled participants from defined populations and measured the internal milieu, which differentiates them from studies of clinic referrals that examine only clinical phenotypes.

**Methods:**

This analysis evaluated 386 women identified in a population-based survey of chronic fatigue and unwellness in metropolitan, urban, and rural populations of the state of Georgia, USA. We used variables previously demonstrated to effectively delineate endophenotypes in an attempt to replicate identification of these endophenotypes. Latent class analyses were used to derive the classes, and these were compared and contrasted to those described in the previous study based in Wichita, Kansas.

**Results:**

We identified five classes in the best fit analysis. Participants in Class 1 (25%) were polysymptomatic, with sleep problems and depressed mood. Class 2 (24%) was also polysymptomatic, with insomnia and depression, but participants were also obese with associated metabolic strain. Class 3 (20%) had more selective symptoms but was equally obese with metabolic strain. Class 4 (20%) and Class 5 (11%) consisted of nonfatigued, less symptomatic individuals, Class 4 being older and Class 5 younger. The classes were generally validated by independent variables. People with CFS fell equally into Classes 1 and 2. Similarities to the Wichita findings included the same four main defining variables of obesity, sleep problems, depression, and the multiplicity of symptoms. Four out of five classes were similar across both studies.

**Conclusion:**

These data support the hypothesis that chronic medically unexplained fatigue is heterogeneous and can be delineated into discrete endophenotypes that can be replicated. The data do not support the current perception that CFS represents a unique homogeneous disease and suggests broader criteria may be more explanatory. This replication suggests that delineation of endophenotypes of CFS and associated ill health may be necessary in order to better understand etiology and provide more patient-focused treatments.

## Introduction

Chronic fatigue syndrome (CFS) is a common, debilitating illness whose hallmark symptoms involve fatigue and fatigability [1-5]. CFS has no diagnostic clinical signs or laboratory markers and is diagnosed based on self-reported symptoms and the ruling out of medical and psychiatric conditions that present similarly. There are several published definitions of CFS [1,4,5] that have proved useful in standardizing research subjects but lack empirical support [6,7]. Several studies have described the heterogeneity of CFS [6,8-11], but they recruited patients from clinical practices and relied on clinical and demographic information rather than physiological data. These studies have failed to identify consistent subgroups. Despite its prevalence and documented heterogeneity, systematic approaches to identify the endophenotypes comprising CFS have not been adopted.

We recently reported a more comprehensive approach to delineate the heterogeneity of medically unexplained chronic fatigue in 159 women from a population-based sample in Wichita, Kansas, USA [12,13]. That study used principal components analysis to screen about 500 clinical, demographic, and laboratory measurements acquired from the 159 women during a two-day in-hospital case-control study and found that 38 variables accounted for the majority of the variance. Latent class analysis of these 38 variables identified four classes as the best fit model. Classes containing the most severely fatigued women were differentiated by sympathetic nervous system and endocrine activity, polysomnographic measures of sleep, mood disturbance, and multiplicity of symptoms. Women in Class 1 (32%) were unwell and obese with laboratory findings characteristic of metabolic strain. They had polysomnographic changes of hypnoea and were depressed and polysymptomatic. Women in Class 2 (28%) were obese but well. Those in Class 3 (26%) were unwell, polysymptomatic, and depressed, but had relatively normal body mass indices and normal biological markers. Finally, Class 4 (16%) comprised relatively well, non-obese women who were more symptomatic than Class 2.

We validated these latent classes against various independent measures, including severity, disability, gene expression profiles, and single nucleotide polymorphisms [13-15]. This is in accord with results from another study of 55 patients recruited from specialist care that has reported that quantitative gene expression analysis can differentiate seven CFS subgroups [16]. However, only gene expression was used to delineate the subgroups.

The objective of the current analysis was to replicate our previous comprehensive delineation approach with data collected during a survey of people from a different population, identified from metropolitan, urban, and rural populations of Georgia [17]. Although there were some differences in the measures used, we hypothesized that we would confirm the heterogeneity of medically unexplained chronic fatigue, and that the same measures would differentiate similar subgroups as those defined in the Wichita population-based study.

## Methods

This study adhered to human experimentation guidelines of the U.S. Department of Health and Human Services and the Helsinki Declaration. The CDC Institutional Review Board approved the study protocol. All participants were volunteers who gave informed consent.

### Study participants

**Figure 1:**
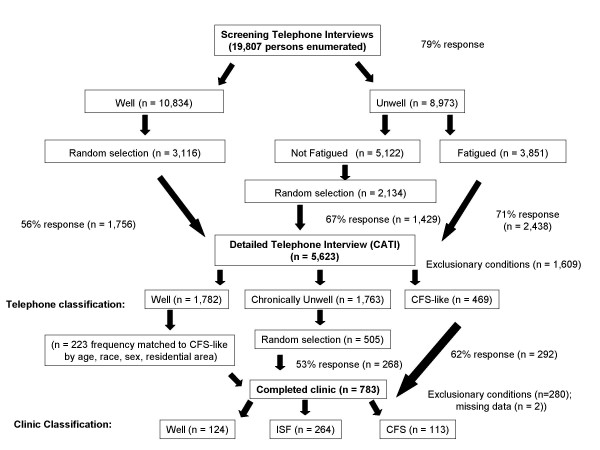
**Metropolitan, Urban, and Rural Georgia Study Populations**.

This study was part of a larger effort to evaluate the occurrence of, and risk factors for, CFS and unwellness in the 18- to 59-year-old population of the state of Georgia. Figure 1 summarizes the sample, and details are available elsewhere [17]. Briefly, between September 2004 and July 2005, we used random digit dialing to conduct a household screening interview with a household informant in three Georgia populations (metropolitan, urban, and rural). A household informant described demographics and health status of household members ages 18 to 59. That initial interview enumerated 19,807 adult residents and screened for unwellness among household members for whom at least one CFS symptom was reported (fatigue, impaired cognition, un-refreshing sleep, muscle or joint pain). Well residents had none of these symptoms for ≥ one month. The screening interview identified 10,834 (55%) well people; 5,122 (26%) people who were unwell for at least a month but not fatigued; and 3,851 (19%) people who were unwell and fatigued for at least a month. We then conducted detailed telephone interviews with all those identified as unwell with fatigue, a random selection of those who were unwell but without fatigue, and a random sample of well people (see Figure 1). Based on their responses to the detailed telephone interview, we classified participants as CFS-like if they met criteria of the 1994 CFS case definition [5]; as chronically unwell if they exhibited some but not all CFS symptoms; and as well if they reported no such symptoms. Finally, we invited all 469 people classified as CFS-like; 641 well people (matched to the CFS-like people by sex, race/ethnicity, age, and geographic stratum); and a similar number (n = 505) of randomly selected unwell people for a one-day clinical evaluation. Overall, 48.5% completed the clinical evaluation.

### Clinical evaluation and illness classification

To identify medical conditions considered exclusionary for CFS [5,7], the clinical evaluation included a standardized past medical history, a review of systems, a standardized physical examination, and routine laboratory testing of blood and urine [5,7]. To identify psychiatric conditions considered exclusionary for CFS, licensed and specifically trained psychiatric interviewers administered the Structured Clinical Interview for DSM-IV (SCID) [18]. Exclusionary psychiatric disorders included current melancholic major depression, any psychotic condition, bipolar affective disorder, active substance abuse/dependence, anorexia or bulimia nervosa [5,7]. Subjects with current non-melancholic major depressive episode or a past history of major depressive disorder were not excluded. The study identified exclusionary medical or psychiatric conditions in 280 (36%) of those evaluated clinically. They were excluded from further analyses, as were two others with incomplete data, leaving a total sample of 501 subjects.

We also diagnosed CFS based on the clinical evaluation according to criteria of the 1994 case definition [5] and as recommended by the International CFS Study Group [7], which is standard in CDC studies of CFS. Thus, we evaluated functional impairment by means of the Medical Outcomes Short-Form Health Survey (SF-36) [19]. We used the Multidimensional Fatigue Inventory (MFI-20) [20] to measure characteristics of fatigue, and we utilized the CDC CFS Symptom Inventory to document occurrence, frequency, and severity of the defining symptoms [21]. Subjects who had ≥ four case-defining symptoms, who exceeded the Symptom Inventory cut-off score, and who met CFS cut-off scores on the SF-36 and the MFI-20 were considered to have CFS (n = 113 participants). Those who met at least one, but not all CFS criteria, comprised the ISF group (n = 264), and those who met none of the cut-off criteria comprised the Well group (n = 124). In consideration of inadequate sample size for detecting the heterogeneity in both sexes, and for replication of the Wichita study (which only studied women), we opted to only study women. This left 386 women in the analysis for this paper.

### Independent variables used to delineate the subgroups

Although many of the 38 variables used in the Wichita study were the same (see Table 1), some, such as nocturnal polysomnography, were not measured in the Georgia study. In all, 26 clinical and laboratory variables were the same or very similar to the 38 variables used in the Wichita study [13]. When a variable was not available, we used proxy measures to estimate the effect of the missing variable (see Table 1). Cortisol index was the sum of four serum cortisol concentrations taken regularly over 24 hours minus the baseline concentration.

**Table 1:** Defining variables used in the Wichita and Georgia studies

**Wichita**	**Georgia**
Unrefreshing sleep	Unrefreshing sleep

Sleep problems	Sleep problems

Post-exertional fatigue	Post-exertional fatigue

Muscle pain	Muscle pain

Joint pain	Joint pain

Severe headache	Headache

Concentration	Concentration

Photophobia	Photophobia

Sore throat	Sore throat

Shortness of breath	Shortness of breath

Stomach or abdominal pain	Stomach or abdominal pain

Fever	Fever

Nausea	Nausea

Depression (Zung score)	Depression (Zung score)

Sleepiness (Epworth score)	Sleepiness (Epworth score)

O_2_saturation	

Sleep resp. disturbance index	

Sleep-total time	

Sleep-total arousals	

Sleep-HR variability	Heart rate at rest

Sleep REM latency	

Age	Age

Body mass index	Body mass index

Neck circumference	Neck circumference

High sensitivity CRP	High sensitivity CRP

Serum insulin	Serum insulin

Free T3	

Progesterone	

Free testosterone	

24 hours Urinary free cortisol	Cortisol index

Hemoglobin	Hemoglobin

Bilirubin	Bilirubin

AST	AST

IL-6	

IL-1b	

Igf-1	

Tilt BP_5min_stand_index	Systolic blood pressure

### Independent variables used to validate the subgroups

In order to externally validate the subgroups, we used the Short-Form Health Survey measure of functioning (SF-36) [19] and the Multidimensional Fatigue Inventory [20], neither of which had been used to define the classes.

### Statistical analysis

We used Latent GOLD^® ^(LatentGOLD 4.1.3, Statistical Innovations, MA, USA) software to perform latent class analyses (LCA) on all variables and all subjects. Non-categorical variables were dichotomized by median split, and we filled missing (null) values (0.4% missing data values) with an intermediate categorical value. LCA is a statistical method for finding subtypes of similar subjects (latent classes) using multiple, defining, and categorical variables. LCA searches for an optimal class assignment for each subject so that most of the variation in the entire dataset can be explained by the subject's class assignment alone. LCA posits the existence of statistically uncorrelated measurements within each of the discovered classes. One therefore seeks measurements that most fully define the variability in the dataset, but are uncorrelated with each other. LCA and other clustering techniques seek to find loci in the multidimensional space of measurements (clinical and biological) where subjects cluster together. The subjects that are close to each other belong to the same class. Finding the locus for each class requires fitting one parameter for each dimension (measurement), and specifying a locus in an n-dimensional space requiring n parameters. To determine the optimal number of latent classes, we utilized the Akaike Information Criterion (with 3 as penalizing factor) (AIC3). AIC3 is defined by the following equation:



where *L *is the likelihood and *npar *is the number of estimated parameters.

Recent results [22,23] suggest AIC3 is a better criterion than Bayesian or AIC for LCA models. We utilized AIC3 for the Wichita study and also utilize it here. The optimal number of classes is determined through locating a minimum in the value of AIC3 for solutions with different numbers of clusters. To validate the different classes, we compared independent variables across the empirically defined classes using Kruskal-Wallis or Mann-Whitney U tests for interval variables. Variables tested for validity included the Short-Form Health Survey (SF-36) and the Multidimensional Fatigue Inventory. A final test of validation was used to compare the empirically defined classes against a standardized application of impairment, fatigue, and symptom criteria of the 1994 CFS case definition [24] as recommended by the International CFS Study Group [7] and the screening criteria of "unwell and not fatigued" and "well."

## Results

The AIC3 results for the four-, five-, and six-class solutions were respectively 13,856, 13,844, and 13,846. A five-class solution was statistically most significant, as the AIC3 was the lowest for this solution (13,844), and we therefore only show these data (four- and six-class solutions not shown).

Table 2 shows the five classes and associated data relating the variables that contributed significantly to the model as indicated by the Wald statistic. Class 1 (25%) captured ill subjects with many symptoms, prominent fatigue, sleep problems, and depression, but no aberrant biological markers (including body mass index) characterized this group. Class 2 (24%) similarly captured ill subjects who reported prominent, widespread symptoms, insomnia, and depression. However, these subjects had an associated metabolic syndrome (elevated insulin and inflammatory markers) and were obese. Class 3 (20%) included less ill subjects with fewer symptoms but similarly obese individuals with elevated serum insulin and inflammatory markers. Class 4 (20%) and Class 5 (11%) primarily included well individuals of normal weight. Class 5 included the youngest individuals and Class 4 the oldest.

**Table 2:** Latent classes obtained from a five-class solution

**Class Attributes**	**Class-1**	**Class-2**	**Class-3**	**Class-4**	**Class-5**	**Wald**	**p-Value**
Number (%) of subjects	**97 (25)**	**92 (24)**	**77 (20)**	**76 (20)**	**44 (11)**		

Stomach problems (%)	**57**	**46**	**25**	**12**	**18**	**39.2**	**0.000**

Concentration difficulty	**33**	**53**	**5**	**0**	**0**	**29.1**	**0.000**

Fever	**22**	**28**	**6**	**7**	**16**	**17.6**	**0.002**

Headache	**84**	**79**	**53**	**46**	**77**	**33.8**	**0.000**

Joint pain	**61**	**71**	**34**	**30**	**0**	**34.0**	**0.000**

Muscle pain	**81**	**87**	**44**	**46**	**34**	**46.9**	**0.000**

Nausea	**37**	**44**	**10**	**5**	**18**	**31.2**	**0.000**

Photophobia	**44**	**63**	**14**	**8**	**0**	**51.6**	**0.000**

Shortness of breath	**22**	**47**	**8**	**0**	**4**	**32.0**	**0.000**

Sore throat	**52**	**58**	**13**	**13**	**36**	**45.0**	**0.000**

Unusual fatigue	**70**	**86**	**32**	**8**	**11**	**89.6**	**0.000**

Unrefreshing sleep	**98**	**97**	**45**	**41**	**14**	**63.9**	**0.000**

Sleep problems	**94**	**97**	**49**	**46**	**29**	**68.4**	**0.000**

Sleepiness (Epworth score)	**10**	**10**	**5**	**6**	**5**	**58.6**	**0.000**

Depression (Zung score)	**53**	**56**	**43**	**39**	**35**	**92.4**	**0.000**

Age (years)	**42**	**44**	**48**	**51**	**38**	**37.6**	**0.000**

BMI (kg/m^2^)	**24**	**32**	**32**	**24**	**22**	**85.6**	**0.000**

Neck circumference (cm)	**33**	**37**	**37**	**34**	**32**	**72.6**	**0.000**

High sensitivity CRP (mg/dL)	**0.10**	**0.42**	**0.51**	**0.1**	**0.08**	**72.4**	**0.000**

Serum insulin (units?)	**3.9**	**8.9**	**8.4**	**3.9**	**3.7**	**79.4**	**0.000**

Cortisol index	**1.9**	**1.5**	**2.0**	**1.7**	**2.2**	**11.1**	**0.026**

Serum bilirubin	**0.4**	**0.4**	**0.4**	**0.5**	**0.4**	**14.5**	**0.006**

Serum AST	**15**	**16**	**15**	**18**	**15**	**15.9**	**0.003**

Hemoglobin	**13.0**	**13.4**	**13.1**	**13.3**	**12.6**	**20.6**	**0.000**

Heart rate (bpm)	**68**	**68**	**72**	**64**	**64**	**9.8**	**0.043**

Systolic blood pressure	**110**	**124**	**126**	**120**	**108**	**55.2**	**0.000**

Figures show (%) of total number in a class endorsing a symptom, median score on self-rating scales, or for biological markers, the median value for the respective class. The variables shown in the table are those significantly contributing to the class solution according to the Wald statistic. BMI = Body Mass Index. CRP = C reactive protein. AST = Aspartate transaminase.

As in the Wichita study, the four-class solution (data not shown) (AIC3 = 13,856) divided the sample along the dimension of obesity, resulting in two obese and two normal-weight classes. A secondary division on the basis of symptomatology shaped the final four classes such that Class 1 (27% of subjects) constituted a group of well subjects with normal BMI (median BMI = 23; median age = 45). Class 2 (25%) contained symptomatic and depressed subjects, with prominent sleep problems and normal BMI (median BMI = 24, median age = 42) but without abnormal biological markers. Class 3 subjects (25%) were obese (median BMI = 33; median age = 44), with the highest levels of symptoms, prominent depression, and poor sleep. In addition, these subjects had low cortisol levels and showed signs of obesity-associated metabolic strain, including higher levels of insulin and inflammatory markers. Class 4 (23%) consisted of obese older individuals (medium BMI = 31; median age = 48) with some sign of metabolic strain (e.g., raised insulin and inflammatory markers), but who were otherwise well.

### Validation against independent variables not included in the model

The median values on the variables originally omitted from deriving optimal class solutions are shown for each of the five classes in Table 3. With all five classes included in the analysis, nonparametric ANOVA produced highly significant overall tests indicative of substantial between-class differences for all variables (see Table 4). We used Mann-Whitney U follow-up tests for fatigue and disability scores to compare each of the three ill classes against the two well classes (Classes 4 and 5). This confirmed significant differences across all the dimensions of disability and fatigue between Classes 1 and 2 and the well Classes 4 and 5 (all *P *values < 0.001). Comparisons among the less symptomatic but still ill Class 3 and both of the well classes still produced highly significant differences for physical functioning, and for general, physical, and mental fatigue (all *P *values < 0.008). Similarly, there were significant differences in activity and motivation (all *P *values < 0.009). Reported levels of mental and social functioning were substantively different only between Class 3 and Class 5 (*P *values < 0.003).

**Table 3:** Descriptive data, disability, and fatigue scores (as medians) for each class defined in the five-class solution

**Descriptive Data**	**Class-1**	**Class-2**	**Class-3**	**Class-4**	**Class-5**
Number in class	**97**	**92**	**77**	**76**	**44**
	
Age (years)	**42**	**44**	**48**	**51**	**38**
	
CFS illness duration (years)	**4.3**	**2.8**	**5.1**	**N/A**	**N/A**

**Disability (SF-36)**					

Physical Functioning	**80**	**70**	**85**	**93**	**100**
	
Mental Health	**60**	**60**	**84**	**88**	**92**
	
Social Functioning	**75**	**56**	**100**	**100**	**100**

**Fatigue (MFI)**					

General Fatigue	**16**	**16**	**12**	**8**	**7**
	
Physical Fatigue	**12**	**14**	**11**	**7**	**6**
	
Mental Fatigue	**13**	**13**	**8**	**7**	**5**
	
Reduced Activity	**9**	**11**	**7**	**6**	**6**
	
Reduced Motivation	**11**	**12**	**8**	**6**	**6**

SF-36 = Short-Form Health Survey

MFI = Multidimensional Fatigue Inventory

**Table 4:** Independence of all five classes and the three classes defining ill/fatigued subjects

	**All Classes**		**3 Ill Classes**	
**Disability (SF-36)**	**χ^2^**	***P***	**χ^2^**	***P***

Physical functioning	**129.2**	**0.000**	**26.8**	**0.000**
	
Mental health	**133.5**	**0.000**	**48.0**	**0.000**
	
Social functioning	**159.1**	**0.000**	**57.1**	**0.000**

**Fatigue (MFI)**				

General fatigue	**177.8**	**0.000**	**47.1**	**0.000**
	
Physical fatigue	**149.2**	**0.000**	**22.7**	**0.000**
	
Mental fatigue	**164.4**	**0.000**	**64.5**	**0.000**
	
Activity reduction	**80.1**	**0.000**	**17.6**	**0.000**
	
Motivation reduction	**117.4**	**0.000**	**30.2**	**0.000**

SF-36 = Short-Form Health Survey

MFI = Multidimensional Fatigue Inventory

Significant between-class differences also distinguished the three ill/fatigued classes in all disability and fatigue categories (Table 4). Follow-up tests showed no significant differences between Classes 1 and 2 on the SF-36 dimensions [19] of mental functioning and the Multidimensional Fatigue Inventory subscales [20] of general fatigue, mental fatigue, and reduction in motivation (all *P *values > 0.05). However, Class 2 reported significantly lower physical (*P *< 0.000) and social functioning (*P *< 0.01) and higher levels of physical fatigue (*P *< 0.007) and activity reduction (*P *< 0.009) than Class 1. Comparisons of SF-36 and fatigue scores between Classes 1 and 3 revealed no statistically significant difference in SF-36 physical functioning, but differences on the dimensions of mental and social functioning (both *P *values < 0.000). Additional differences between these two classes were evident on general fatigue, mental fatigue, and reduction in motivation (all *P *values < 0.000), and to a lesser degree for physical fatigue (*P *< 0.01) and activity reduction (*P *< 0.04). Comparisons between the most symptomatic Class 2 and the least symptomatic Class 3 of the ill classes were all significantly different at the P < 0.000 level.

Table 5 shows the distribution of criterion-based diagnoses: CFS, unwell but not fatigued, and well individuals. People diagnosed as CFS fell almost exclusively into Classes 1 and 2. Those who were unwell but not fatigued were fairly evenly spread across all classes, while the well were most commonly found in Classes 4 and 5, with a small minority in Class 3.

**Table 5:** Distribution of cases identified by the CDC research criteria for unexplained fatigue (CFS and unwell/not fatigued) and well controls across the empirically derived five classes: N (%)

	**Class 1** **(n = 97)**	**Class 2** **(n = 92)**	**Class 3** **(n = 77)**	**Class 4** **(n = 76)**	**Class 5** **(n = 44)**
CFS (n = 92)	41 (44)	47(51)	3(3)	1(1)	0

unwell not fatigued (n = 201)	53 (26)	43(21)	56(28)	32(16)	17(8)

well (n = 93)	3 (3)	2(2)	18 (19)	43 (46)	27 (29)

Table 6 compares and contrasts the four class solutions across the two studies in Georgia and Wichita. There was a close similarity in the content of all four classes. Table 7 compares and contrasts the five class solutions across the same two studies. Four out of the five classes were very similar in their content.

**Table 6:** Comparisons of four class solutions across the two studies

**Summarized content of classes**	**Georgia Class (% cases)**	**Wichita Class (% cases)**
Not obese and well	1 (27)	4 (16)

Not obese, polysymptomatic, depressed, insomnia	2 (25)	3 (26)

Obese, metabolic strain, polysymptomatic, insomnia, depressed	3 (25)	1 (32)

Obese, metabolic strain, but relatively well	4 (20)	2 (28)

**Table 7:** Comparisons of five class solutions across the two studies

**Summarized content of classes**	**Georgia Class (% cases)**	**Wichita Class (% cases)**
Not obese, polysymptomatic, depressed, insomnia	1 (25)	5 (13)

Obese, metabolic strain, polysymptomatic, depressed, insomnia	2 (24)	1 (28)

Obese, metabolic strain, fewer symptoms of pain and insomnia, not depressed	3 (20)	2 (23)

Not obese, fewer symptoms of pain and insomnia, not depressed	4 (20)	4 (17)

Well, bar headache, young	5 (11)	

Obese, metabolic strain, fewer symptoms of pain and insomnia, depressed		3 (19)

## Discussion

This analysis supported the heterogeneity of unexplained chronic fatigue in a population-derived sample. The most statistically rigorous latent classes were five in number, although the four-class solution was equally interpretable. The five-class solution provided three ill classes and two relatively well classes. The ill classes were differentiated by multiple symptoms, obesity, metabolic strain, depressed mood, and sleep problems. The four-class solution provided two well and two unwell groups, with differentiating variables being the same as for the five-class solution. The validation of the classes was supported by the significant differences across classes in the independent variables, such as fatigue subscale scores and disability. CFS was divided across two subgroups, while the unwell but not fatigued group was more heterogeneous, represented in all five classes. As expected, well people primarily made up Classes 4 and 5.

### Comparison with the Wichita study

How do these results compare with the Wichita classes? First, the main variables that differentiated the classes were similar: obesity, metabolic strain, multiplicity of physical symptoms, sleep problems, and depression. Secondly, the four-class solutions were very similar across the two studies, and four out of the five class solutions were also similar.

The dissimilarities include empirically describing a five-class solution in Georgia compared to a four-class solution in Wichita. The absence of measures of sympathetic nervous system arousal, such as nocturnal heart-rate variability and polysomnography, used in the Wichita study, may explain the lack of further similarities between the two samples. Similarly, the inability to differentiate a sixth class, similar to that reported in the Wichita sample, was probably related to the absence of the sex steroid hormone assays in the Georgian sample because the sixth class was defined primarily by being post-menopausal [12]. The SF-36 physical function subscale scores were higher than we expected, but the scores were similar to those of the relevant subjects from the Wichita study and probably reflect the sample being derived from the population rather than a clinic. Previous studies of CFS have also used a previous version (version 1 rather than 2) of the SF-36 scale, with a slightly different stem question, which also may have affected the data.

### Comparisons with other studies of heterogeneity

The multiplicity of symptoms in some classes supports the two previous studies differentiating CFS on the basis of a minority having features of somatisation [8,9]. The importance of depression reflects the differentiation found on the basis of mood disturbance in the largest study of heterogeneity using the Swedish twin registry, which found two main subgroups, one of which was differentiated primarily by comorbid mood disorders [25]. Kerr and colleagues' finding of seven subgroups was based on secondary and tertiary care samples and was determined by gene expression alone [16]. Apart from the Wichita study, for which this is a replication, no other study has defined subgroups using both clinical and laboratory data, so no other comparisons are possible.

### Limitations of this replication

The most important limitation was the absence of some variables that had been used in the Wichita study. These primarily included objective measures of sleep, such as polysomnography, and autonomic nervous system tests such as heart rate variability. Our employment of proxy variables was unsuccessful because they rarely played a differentiating role in the analyses. We were careful to otherwise keep the variables and their preliminary processing, as well as the analyses, the same. In view of the discrepancy in important variables, it is remarkable that so many similarities were found. The addition of other variables, which neither study measured, such as actigraphy and other psychiatric comorbidities (e.g., anxiety), might have further enhanced the differentiation of classes [26]. As in all population surveys, there may have been a response bias of which we were not aware. Finally, this replication was based on American women, as in the Wichita study, so that these data cannot necessarily be generalized across other nations or to men.

## Conclusion

### Implications of this replication

What are the clinical implications of this work? This study's support for the heterogeneity of CFS, found in an independent population-derived sample, adds to the weight of evidence for the heterogeneous nature of CFS. We do not yet know whether this heterogeneity is important in determining response to different treatments, but future research studies should now examine for moderators of outcome that include obesity, metabolic syndrome, sleep problems, depression, and having multiple symptoms.

As in Wichita, these data suggest that the current research criteria for CFS, while useful in providing reliable findings across studies, do not constitute a homogeneous group of patients. The broadening of the concept of CFS to include patients with fewer symptoms but similar disability is supported by this replication [6,7].

What are the research implications of this work? Future etiological work needs to take into account the heterogeneity of CFS, using the determining variables, such as obesity, multiple symptoms, and depression, to stratify samples. For instance, obesity leads to fatigue in multiple ways, including sleep disturbance, metabolic strain, increased inflammatory markers, and deconditioning [12,27,28]. This heterogeneity may well explain the dearth of replicated etiological findings in CFS. Addressing heterogeneity up front may help to determine the different etiologies and pathophysiologies that likely determine the different illnesses that make up CFS.

## Competing interests

The authors declare that they have no competing interests.

## Authors' contributions

PW provided study concept and design. UVC and PW provided clinical and biological expertise. WR conceived, originated, and executed the Georgia study, from which the data originated. WR also provided expert advice in data interpretation. EA provided data analysis. All authors participated in the writing and editing of the manuscript. All authors read and approved the final manuscript.

## Disclaimer

The findings and conclusions in this report are those of the authors and do not necessarily represent the views of the funding agency.

## References

[B1] Lloyd AR, Hickie I, Boughton CR, Spencer O, Wakefield D (1990). Prevalence of chronic fatigue syndrome in an Australian population. Med J Aust.

[B2] Jason LA, Richman JA, Rademaker AW, Jordan KM, Plioplys AV, Taylor RR, McCready W, Huang CF, Plioplys S (1999). A community-based study of chronic fatigue syndrome. Arch Intern Med.

[B3] Reyes M, Nisenbaum R, Hoaglin DC, Unger ER, Emmons C, Randall B, Stewart JA, Abbey S, Jones JF, Gantz N, Minden S, Reeves WC (2003). Prevalence and incidence of chronic fatigue syndrome in Wichita, Kansas. Arch Intern Med.

[B4] Sharpe MC, Archard LC, Banatvala JE, Borysiewicz LK, Clare AW, David A, Edwards RHT, Hawton KEH, Lambert HP (1991). A report--chronic fatigue syndrome: guidelines for research. J Roy Soc Med.

[B5] Fukuda K, Straus SE, Hickie I, Sharpe MC, Dobbins JG, Komaroff A (1994). The chronic fatigue syndrome: a comprehensive approach to its definition and study. International Chronic Fatigue Syndrome Study Group. Ann Intern Med.

[B6] Sullivan PF, Pedersen NL, Jacks A, Evengard B (2005). Chronic fatigue in a population sample: definitions and heterogeneity. Psychol Med.

[B7] Reeves WC, Lloyd A, Vernon SD, Klimas N, Jason LA, Bleijenberg G, Evengard B, White PD, Nisenbaum R, Unger ER, International Chronic Fatigue Syndrome Study Group (2003). Identification of ambiguities in the 1994 chronic fatigue syndrome research case definition and recommendations for resolution. BMC Health Serv Res.

[B8] Hickie I, Lloyd A, Hadzi-Pavlovic D, Parker G, Bird K, Wakefield D (1995). Can the chronic fatigue syndrome be defined by distinct clinical features?. Psychol Med.

[B9] Wilson A, Hickie I, Hadzi-Pavlovic D, Wakefield D, Parker G, Straus SE, Dale J, McCluskey D, Hinds G, Brickman A, Goldenberg D, Demitrack M, Blakely T, Wessely S, Sharpe M, Lloyd A (2001). What is chronic fatigue syndrome? Heterogeneity within an international multicentre study. Aust N Z J Psychiatry.

[B10] Sullivan PF, Smith W, Buchwald D (2002). Latent class analysis of symptoms associated with chronic fatigue syndrome and fibromyalgia. Psychol Med.

[B11] Jason LA, Taylor RR, Kennedy CL, Jordan KM, Song S, Johnson D, Torres-Harding S (2003). Chronic fatigue syndrome: symptom subtypes in a community based sample. Women Health.

[B12] Vollmer-Conna U, Aslakson E, White PD (2006). An empirical delineation of the heterogeneity of chronic unexplained fatigue in women. Pharmacogenomics.

[B13] Aslakson E, Vollmer-Conna U, White PD (2006). The validity of an empirical delineation of heterogeneity in chronic unexplained fatigue. Pharmacogenomics.

[B14] Carmel L, Efroni S, White PD, Aslakson E, Vollmer-Conna U, Rajeevan MS (2006). Gene expression profile of empirically delineated classes of unexplained chronic fatigue. Pharmacogenomics.

[B15] Smith AK, White PD, Aslakson E, Vollmer-Conna U, Rajeevan MS (2006). Polymorphisms in genes regulating HPA axis associated with empirically delineated classes of unexplained chronic fatigue. Pharmacogenomics.

[B16] Kerr JR, Petty R, Burke B, Gough J, Fear D, Sinclair LI, Mattey DL, Richards SC, Montgomery J, Baldwin DA, Kellam P, Harrison TJ, Griffin GE, Main J, Enlander D, Nutt DJ, Holgate ST (2008). Gene expression subtypes in patients with chronic fatigue syndrome/myalgic encephalomyelitis. J Infect Dis.

[B17] Reeves WC, Jones JF, Maloney E, Heim C, Hoaglin DC, Boneva RS, Morrissey M, Devlin R (2007). Prevalence of chronic fatigue syndrome in metropolitan, urban, and rural Georgia. Popul Health Metr.

[B18] First MB, Spitzer RL, Gibbon M, Williams JBW (2002). Structured Clinical Interview for DSM-IV-TR Axis I Disorders, Research Version.

[B19] Ware JE (2000). SF-36 health survey update. Spine.

[B20] Smets EM, Garssen B, Bonke B, De Haes JC (1995). The Multidimensional Fatigue Inventory (MFI) psychometric qualities of an instrument to assess fatigue. J Psychosom Res.

[B21] Wagner D, Nisenbaum R, Heim C, Jones JF, Unger ER, Reeves WC (2005). Psychometric properties of the CDC Symptom Inventory for assessment of chronic fatigue syndrome. Population Health Metrics.

[B22] Andrews RL, Currim IS (2003). A comparison of segment retention criteria for finite mixture logit models. Journal of Marketing Research.

[B23] Dias JG Finite Mixture Models: Review, Applications, and Computer-intensive Methods. PhD Dissertation.

[B24] Reeves WC, Wagner D, Nisenbaum R, Jones JF, Gurbaxani B, Solomon L, Papanicolaou DA, Unger ER, Vernon SD, Heim C (2005). Chronic fatigue syndrome--a clinically empirical approach to its definition and study. BMC Medicine.

[B25] Kato K, Sullivan PF, Evengard B, Pedersen NL (2008). A population-based twin study of functional somatic syndromes.

[B26] Prins JB, Meer JW van der, Bleijenberg G (2006). Chronic fatigue syndrome. Lancet.

[B27] Lim W, Hong S, Nelesen R, Dimsdale JE (2005). The association of obesity, cytokine levels and depressive symptoms with diverse measures of fatigue in healthy subjects. Arch Intern Med.

[B28] Resnick HE, Carter EA, Aloia M, Philips B (2006). Cross-sectional relationship of reported fatigue to obesity, diet, and physical activity: results from the third national health and nutrition examination survey. Journal of Clinical Sleep Medicine.

